# Constructing multifunctional solid electrolyte interface via in-situ polymerization for dendrite-free and low N/P ratio lithium metal batteries

**DOI:** 10.1038/s41467-020-20339-1

**Published:** 2021-01-08

**Authors:** Dan Luo, Lei Zheng, Zhen Zhang, Matthew Li, Zhongwei Chen, Ruiguang Cui, Yanbin Shen, Gaoran Li, Renfei Feng, Shaojian Zhang, Gaopeng Jiang, Liwei Chen, Aiping Yu, Xin Wang

**Affiliations:** 1 Guangdong Provincial Key Laboratory of Nanophotonic Functional Materials and Devices, School of Information and Optoelectronic Science and Engineering & International Academy of Optoelectronics at Zhaoqing, South China Normal University, Guangdong, 510006 China; 2 Department of Chemical Engineering, Waterloo Institute of Nanotechnology, University of Waterloo, Waterloo, ON N2L 3G1 Canada; 3 i-LAB, and Vacuum Interconnected Nanotech Workstation (Nano-X), Suzhou Institute of Nano-Tech and Nano-Bionics, Chinese Academy of Sciences, 215123 Suzhou Jiangsu, China; 4 Canadian Light Source, Saskatoon, SK S7N 0X4 Canada; 5 College of Energy, Xiamen University, Xiamen, 361005 China; 6 In-situ Center for Physical Sciences, and Frontiers Science Center for Transformative Molecules, School of Chemistry and Chemical Engineering, Shanghai Jiao Tong University, Shanghai, 200240 China; 7 South China Academy of Advanced Optoelectronics, South China Normal University, Guangdong, 510631 China

**Keywords:** Chemistry, Materials science

## Abstract

Stable solid electrolyte interface (SEI) is highly sought after for lithium metal batteries (LMB) owing to its efficient electrolyte consumption suppression and Li dendrite growth inhibition. However, current design strategies can hardly endow a multifunctional SEI formation due to the non-uniform, low flexible film formation and limited capability to alter Li nucleation/growth orientation, which results in unconstrained dendrite growth and short cycling stability. Herein, we present a novel strategy to employ electrolyte additives containing catechol and acrylic groups to construct a stable multifunctional SEI by in-situ anionic polymerization. This self-smoothing and robust SEI offers multiple sites for Li adsorption and steric repulsion to constrain nucleation/growth process, leading to homogenized Li nanosphere formation. This isotropic nanosphere offers non-preferred Li growth orientation, rendering uniform Li deposition to achieve a dendrite-free anode. Attributed to these superiorities, a remarkable cycling performance can be obtained, i.e., high current density up to 10 mA cm^−2^, ultra-long cycle life over 8500 hrs operation, high cumulative capacity over 4.25 Ah cm^−2^ and stable cycling under 60 °C. A prolonged lifespan can also be achieved in Li-S and Li-LiFePO_4_ cells under lean electrolyte content, low N/P ratio or high temperature conditions. This facile strategy also promotes the practical application of LMB and enlightens the SEI design in related fields.

## Abstract (web summary)

Stable solid electrolyte interface (SEI) is heavily investigated due to its role in improving lithium metal batteries. Here, the authors present a new strategy by employing electrolyte additives to construct stable multifunctional SEI via in situ anionic polymerization.

## Introduction

High-energy-density storage systems are highly imperative to satisfy the ever-increasing energy demand in portable electronics, electric vehicles, and grid-scale storage^1–3^. However, the energy density of existing batteries remains insufficient for many of these applications^4,5^. Lithium (Li) metal is a promising anode material for high-energy-density storage systems owing to its high specific capacity (3860 mAh g^−1^) and the lowest reduction potential (−3.04 V vs. standard hydrogen electrode)^6,7^. Despite these unique advantages, the development of practical rechargeable LMB is still hindered by several technical issues. The highly reactive Li metal has spontaneous reaction with electrolyte, leading to solid electrolyte interface (SEI) layer formation on Li’s surface, which blocks the electrolyte contact and inhibits its reduction. However, this shaped SEI is unstable with poor mechanical properties, which could continuously break and accumulate during dynamic Li plating/stripping process, leading to ever-increased polarization upon cycling^8–10^. The uneven current/ion distribution and the texture formation can result in non-uniform Li deposition/growth along the preferred orientation, which further exacerbates the Li dendrite formation, resulting in low Coulombic efficiency (CE), poor cycle life span and severe safety hazards of batteries^11,12^. In addition, the cathode chemistry also has substantial influence on the SEI stability^13,14^. In the lithium–sulfur (Li–S) battery system, highly dissolved lithium polysulfide (LPS) can migrate to anode and spontaneously react with lithium metal, causing irreversible capacity loss of lithium metal anode (LMA)^15^. These issues are more critical under the commercially relevant condition of LMB, such as low negative/positive electrode capacity (N/P) ratio, lean electrolyte content, and extreme temperature conditions^16^. Under these scenarios, most of the original problems, such as fast electrolyte “drying” and capacity fading are amplified^17,18^. Therefore, the realization of high-energy-density LMB, especially Li–S batteries, with satisfying life span under low N/P ratio (≤1.5) and high temperature (≥60 °C) is still hindered toward practical application^19^. It is essential to construct multifunctional SEI with robust structure, excellent mechanical properties, lean electrolyte consumption as well as the ability to regulate Li^+^ flow and suppress Li dendrite growth.

Numerous efforts, such as employing electrolyte additives, designing artificial SEI layer, developing host structure, introducing high concentration electrolyte and optimizing electrolyte component, have been attempted to construct a stable SEI towards improved LMB performance^20–22^. However, scarcely strategies have been developed which can simultaneously improve the performance of SEI and tackle all challenges remaining in LMB. Although artificial SEI design delivers a promising solution to shape dendrite-free LMA, it involves tedious fabrication process, and more importantly, the formation of a non-smooth thick layer greatly increases the interfacial resistance^23,24^. Employing electrolyte additives serves as another promising proposition owing to its ease to scale up. However, the introduction of additives may induce texture formation during Li plating, resulting in Li dendrite growth along preferred orientation^25,26^. Thus, developing a feasible solution to enable multifunctional SEI formation is the key factor to realize dendrite-free and long cycle life LMA.

Interestingly, in nature, the extensible byssus of mussel could attach to almost all types of organic or inorganic surfaces owing to the strong adsorption of catechol family molecules^27,28^. Introducing catechol as SEI component may endow SEI with strong chemical interaction to reduce electrolyte decomposition^29,30^. Besides, the unsaturated carbon atom covalently linked to electron-withdrawing carboxylic group is highly active for anionic polymerization^31^. It is reported that anionic polymerization of acrylates can be initiated by Li metal, conferring a favorable polymeric layer formation on LMA^32^. Therefore, designing advanced electrolyte additives that synegistically combine the functionalities of catechol and acrylic groups may give rise to a stable multifunctional SEI, fulfilling the requirement for high-energy-density LMB application.

Herein, a novel strategy which employs functional additives containing catechol and acrylic groups to shape multifunctional SEI is developed to realize dendrite-free, long-life and low N/P ratio LMB application. Caffeic acid (CA) was selected as the representative additive. An organic film is established upon the CA anionic polymerization, which further encompasses lithium salt and inorganic Li component as the SEI layer. The multiple hydrogen bonding in the polymeric film offers strong interaction with electrolyte to inhibit its decomposition. Meanwhile, this soft and robust SEI is capable of regulating the morphology and structure to obtain a uniform spherical nanosized Li deposition with non-preferred orientation. This structure evolution of LMA and its substantial impact on achieving dendrite-free LMA is further elucidated by synchrotron grazing-incidence X-ray diffraction (GIXD) and density functional theory (DFT) calculations. Therefore, the multifunctional SEI can conform to Li’s surface morphology and regulate Li deposition, leading to extremely low polarization and efficient Li dendrite formation/growth inhibition. Attributed to these superiorities, the LMA demonstrates remarkable electrochemical performance, i.e., stable operation under high current density up to 10 mA cm^−2^, excellent cycling stability under high temperature condition, and ultra-long cycling life over 8500 h with a high cumulative capacity of 4.25 Ah cm^−2^. When paired with sulfur cathode, an enhanced Li–S full-cell performance was achieved under high sulfur loading, low N/P ratio of ~1.5 and decreased electrolyte/sulfur (E/S) ratio. Besides, an improved cycling performance can be realized in Li–LiFePO_4_ full cell under low N/P ratio of ~2 and lean electrolyte content of 6 g Ah^−1^. This facile and universal strategy offers a new route to realize the practical application of high-energy-density LMB and sheds light on the development of SEI design strategies in other related areas.

## Results

### Formation of multifunctional SEI

**Fig. 1:**
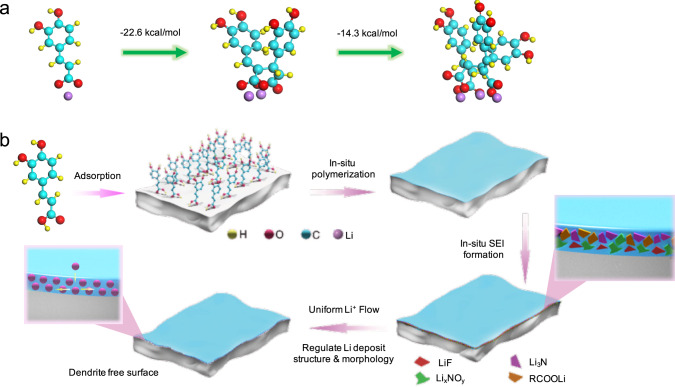
Schematic diagram of SEI formation. **a** The free energies of polymerization process. **b** Schematic illustration of multifunctional SEI formation on LMA.

The CA molecule exhibits a high-binding energy (BE) on Li, indicating its strong chemical interaction with Li metal by forming LC on the surface (Supplementary Fig. 1). The adsorbed lithium caffeinate (LC) molecule is capable of being anionic polymerized initially by Li (Supplementary Fig. 2). One electron transfers from metal Li to the unsaturated C=C double bonds and subsequently initializes the chain propagation. This induces a mild polymerization reaction, resulting in the formation of thin polymer film (noted as CA–Li) on Li’s surface. Reaction free energies were further evaluated for CA–Li polymerization, which demonstrate negative energies during reaction, indicating its thermodynamic favored polymerization process (Fig. 1a). Figure 1b illustrates the shape process of surface layer via adsorption, polymerization, and SEI formation. The scanning electron microscopy (SEM) reveals the uniform coverage of CA–Li on LMA. An organic layer can be observed on LMA after dipping electrolyte, confirming the organic layer formation on the surface (Supplementary Fig. 3B). After 7 days immersion in CA–LiNO_3_ electrolyte, the LMA still demonstrates flat surface without damage, indicating the non-corrosive properties of CA–Li (Supplementary Fig. 3C). The electrolyte is further reduced to inorganic Li compound during electrochemical reduction, leading to hybrid SEI formation. The molecular orbitals were probed to check the reduction capability of additives while the molecule with a lower lowest unoccupied molecular orbital (LUMO) is easy to be reduced^33^. The optimal structures of 1,3 dioxolane (DOL), dimethoxyethane (DME), LiNO_3_, bis(trifluoromethane)sulfonimide lithium salt (LiTFSI), CA, LC and corresponding LUMO and highest occupied molecular orbital (HOMO) energy are examined (Supplementary Fig. 4 and Supplementary Table 1). Clearly, CA and LC have lower LUMO energy than DOL and DME, suggesting their favorable reaction capabilities for SEI formation.

**Fig. 2:**
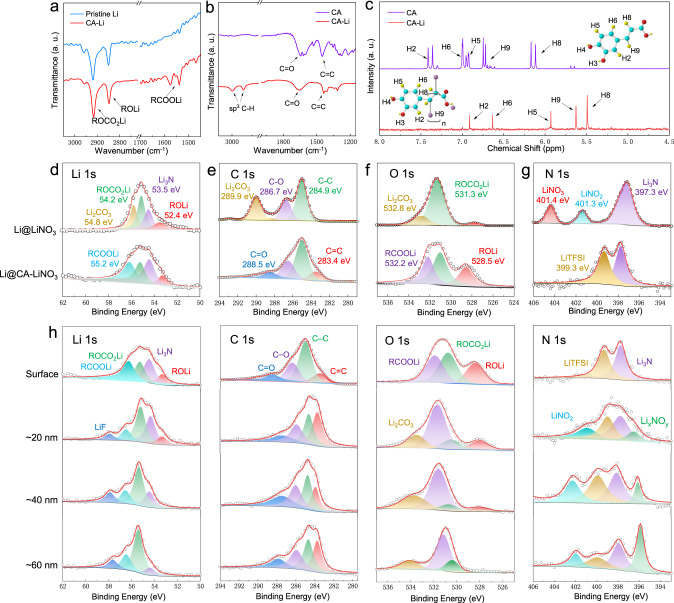
Structure analyzation of SEI. **a** ATR-FTIR spectra of Li with CA-Li, **b** FTIR spectra of CA and CA-Li, **c** ^1^H NMR of CA–Li and CA. High-resolution XPS analyzation of the **d** Li 1*s*, **e** C 1*s*, **f** O 1*s*, and **g** N 1*s* peaks obtained from SEI films of Li@LiNO_3_ (top spectrum) and Li@CA-LiNO_3_ (bottom spectrum). **h** The XPS depth profiles of Li@CA-LiNO_3_ (The in-depth spectra from top to down were collected after Ar sputtering. All the electrodes were prepared after 10th stripping/plating process).

The adsorption of CA on Li metal is confirmed by attenuated total reflection Fourier-transform infrared spectroscopy (ATR-FTIR) spectra (Fig. 2a), which show adsorption peaks at 1469 and 1578 cm^−1^, corresponding to C=C and –COOR content, respectively^30,34^. The peaks located at 2917 and 2848 cm^−1^ can be attributed to the formation of ROCO_2_Li due to the electrolyte reduction^9^. The anionic polymerization of CA–Li is further verified by FTIR and nuclear magnetic resonance (NMR) analysis. Figure 2b demonstrates the FTIR spectra of the CA and CA–Li. As all the carbon atoms in CA molecules are of *sp*^2^ hybridization, the appearance of *sp*^3^ C–H bond indicates the cleavage and polymerization of the C=C double bond by reaction with Li, forming CA–Li^35^. In Fig. 2c, the ^1^H NMR spectrum of CA shows five sets of peaks between 6 and 7.5 ppm, corresponding to the proton on its aromatic ring and double bond^36,37^. Each proton shows a pair of peak, correponding to the *cis*-CA and *trans*-CA. However, the protons in CA–Li only exhibits a single peak, indicating the disappearance of double bond. This spectrum also witnesses a upfield shift, corresponding to its shielding effect attributed to the polymerization^38^. The disappearance of diffraction peaks in CA–Li and the rising of a broaden peak at 25° also indicates its structure change, which could be related to its polymerization (Supplementary Fig. 5). In addition, the hydrogen bonding of CA strengthens the chemical bond of solvent, which induces LiNO_3_ dissociation, as verified by ^7^Li NMR spectrum (Supplementary Fig. 6). The high NO_3_^−^ concentration in electrolyte facilitates the Li_*x*_NO_*y*_-rich SEI formation, ensuring LMA with high stability. Comparing with LC and LiNO_3_, the peak of CA/LiNO_3_ and CA-Li/LiNO_3_ undergoes a negative shift, which is related to the Li^+^ solvation structure change caused by extra LiNO_3_ dissociation^39^. UV–vis spectra of 1 wt% CA, 2 wt% LiNO_3_, 1 wt% CA + 2 wt% LiNO_3_ solution were further collected (Supplementary Fig. 7). The peak located at 295 nm in LiNO_3_ solution can be assigned to the n–π* transition of DME solvent and this peak in CA and CA + LiNO_3_ further shifts toward longer wavelengths, suggesting the existence of hydrogen bonding between solvent and CA^40^. The strengthened interaction of CA endows solvent with strong capabilities to dissociate Li salt into ions. Therefore, the CA additive not only serves as a key component in flexible and soft CA–Li film, but also alters the LiNO_3_ dissociation capability to shape stable hybrid SEI.

To gain more insights into the SEI formation and Li deposition behaviors, electrochemical performances were conducted. The cyclic voltammetry (CV) curves of DOL/DME solutions with/without CA exhibit similar redox current response below 1 V, indicating negligible electrochemical reaction of CA–Li (Supplementary Fig. 8). The oxidation peak above 4 V is related to the CA oxidation. The conventional ether electrolyte exhibits broad reduction peaks below 1.5 V, corresponding to the SEI formation^41^. However, CA electrolyte exhibits a much lower current response, indicating less LiTFSI decomposition. The LiNO_3_ decomposition behaviors in CA–LiNO_3_ electrolyte was further probed. The LiNO_3_ electrolyte shows distinct reduction peaks at 1.3 V while no reduction peaks can be observed in CA–LiNO_3_ electrolyte, confirming the potent suppression of electrolyte decomposition (Supplementary Fig. 9). In Li–Cu cells, the 1st cycle overpotential of Li nucleation in CA–LiNO_3_ electrolyte is lower than that of LiNO_3_ electrolyte in stable deposition states (61 vs. 105 mV), revealing its enhanced kinetics for Li deposition. Meanwhile, the utilization of Li during cycling in CA–LiNO_3_ electrolytes is remarkably promoted with a very high CE.

### Charcaterizations of multifunctional SEI

To identify the chemical composition and structural stability of SEI, the LMA was evaluated after 10th stripping/plating process under the current density of 1 mA cm^−2^ and capacity of 1 mAh cm^−2^ in symmetric cell, using CA–LiNO_3_ and LiNO_3_ electrolyte (noted as Li@CA–LiNO_3_ and Li@LiNO_3_, respectively). X-ray photoelectron spectroscopy (XPS) profiles were collected and analyzed (Fig. 2 and Supplementary Fig. 10). The surface of Li@LiNO_3_ is mainly composed of inorganic Li_3_N, LiF, Li_2_CO_3_ as well as organic ROLi and ROCO_2_Li^42^. However, Li@CA–LiNO_3_ shows strong peaks of RCOOLi and LiTFSI, corresponding to the high CA–Li and TFSI^−^ content in polymeric layer. To further reveal the SEI structure and component during SEI formation, time-of-flight secondary ion mass spectroscopy (TOF-SIMS) analyzations were provided, which offers ultrahigh chemical selectivity and is capable of separately analyzing the individual components of the region of interest. Clearly, a much intensified C_6_H^−^ and CH_2_^−^ signal can be observed in the initial sputtering of Li@CA–LiNO_3_, which indicates that the top part of the SEI layer contains more long chain organic component, corresponding to the formation of CA–Li layer. A much lower F^−^ counts over sputtering can be observed in Li@CA–LiNO_3_, indicating its lower F content than that of Li@LiNO_3_. In addition, the SO_3_^−^, NO^−^, and CO_3_^−^counts can be clearly observed in Li@LiNO_3_ while no distinctive signals of these fragments can be collected in Li@CA–LiNO_3_, corresponding to the inhibited electrolyte decomposition of Li@CA–LiNO_3_. The 3D reconstructed sputtering images further reveal cumulative signals of CH_2_^−^, F^−^, SO_3_^−^, and CO_3_^−^ on cycled anodes, which indicates the formation of thick SEI layer with enriched fluoride, carbonate and sulfonate component in Li@LiNO_3_, confirming the significantly reduced electrolyte decomposition by employing CA as electrolyte additive (Supplementary Fig. 11)^43^. Overall, the XPS analyzation confirms the existence of CA–Li on Li@CA–LiNO_3_ and its capability to inhibit TFSI^−^ and NO_3_^−^ decomposition. The structure and composition distribution of SEI was also performed by depth-dependent XPS analysis (Fig. 2h and Supplementary Fig. 12). The XPS spectra show a gradually decreased content of RCOOLi, ROLi, and increased amount of Li_*x*_NO_*y*_, LiNO_2_, LiF along with the depth of SEI, suggesting the organic–inorganic feature of hybrid SEI^44^. Meanwhile, the –CH_2_–O–CH_2_– and LiTFSI peaks imply the existence of electrolyte in polymeric layer. As a result, the multiple hydrogen bonding of CA–Li offers strong chemical interaction to potently immobilize electrolyte for less consumption on the interface.

### Structure evolution of LMA

**Fig. 3:**
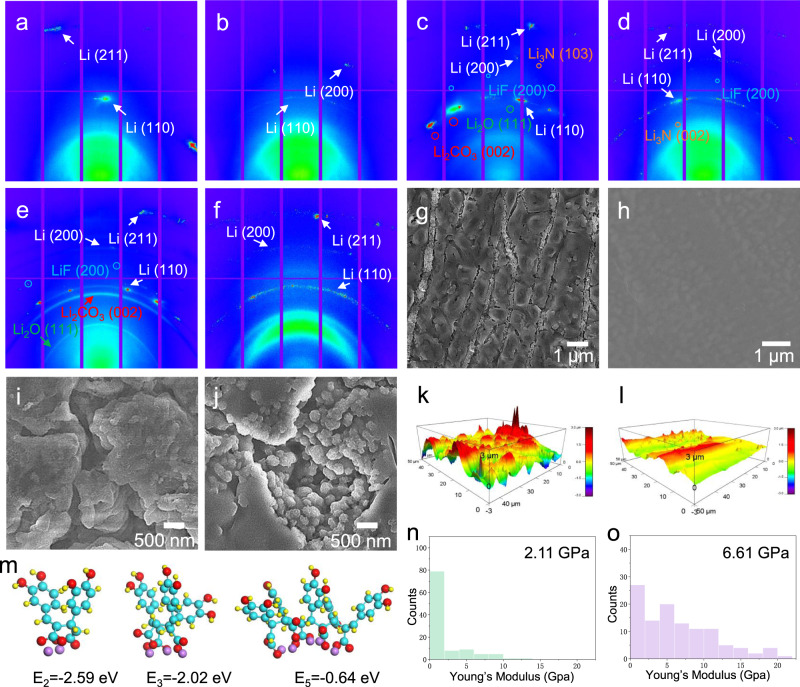
Elucidation of the morphological and structural evolution of LMA. 2D synchrotron grazing-incidence X-ray diffraction (GIXD) pattern of **a**, **c**, **e** Li@LiNO_3_ and **b, d, f** Li@CA–LiNO_3_ after 1st, 10th and 100th stripping/plating process under the current density of 1 mA cm^−2^ with a capacity of 1 mAh cm^−2^. SEM morphology of **g, i** Li@LiNO_3_ and **h, j** Li@CA–LiNO_3_ after 10th and 100th stripping/plating process. AFM image reconstruction of **k** Li@LiNO_3_ and **l** Li@CA–LiNO_3_ after cycling. **m** DFT calculation of geometrical configuration and the binding energy of CA–Li with different chain lengths. Young’s modulus distribution histogram of **n** Li plate and **o** Li@CA–LiNO_3_ after cycling.

The substantial impact of multifunctional SEI on crystal size and morphology regulation was identified by two-dimensional (2D) synchortron GIXD with a beam size of ~3 × 6 μm, which is a new approach to evaluate the dynamic surface structure evolution of LMA. The green-colored region in the GIXD pattern of pristine LMA between 5° and 20° is related to the elastic scattering and Compton scattering (Supplementary Fig. 13). The diffraction spots at 2*θ* angles of 36.19°, 51.97°, and 64.98° can be assigned to the (110), (200), and (211) planes of Li, separately^45^. The corresponding integrated XRD peak reveals its big crystal grains with [211] out-of-plane preferred orientation (Supplementary Fig. 14)^46^. After first stripping, the (211) plane in Li@LiNO_3_ witnesses a significantly reduced peak intensity than (110) and (200) planes, indicating the preferential Li dissolution on (211) plane during stripping process (Supplementary Fig. 15). However, all the peaks in Li@CA–LiNO_3_ present the similar intensity, corresponding to its uniform Li dissolution on each oritentation. After first plating process, the Li@LiNO_3_ shows strong (110) texture formation, indicating the surface energy governed nucleation process (Fig. 3a)^47^. The large diffused XRD spots of Li (110) indicate the strong structural distortion in the deposited Li layer attributed to the non-uniform plating process. Li@CA–LiNO_3_ demonstrates isotropic scattering with multiple discrete XRD spots at 36.2°, confirming the nanorystalline feature of Li deposition (Fig. 3b). After 10th stripping/plating process, more intensive XRD spots related to Li_2_CO_3_, Li_3_N, and LiF can be seen in Li@LiNO_3_ compared with Li@CA–LiNO_3_ (Fig. 3c, d). This result indicates the inhibited side reaction by the multifunctional SEI, which is consistent with the XPS analyzation. A polycrystalline ring-like pattern of Li can be seen in Li@CA–LiNO_3_, confirming the structural evolution of Li from microsized grain to nanocrystals during the acitvation process. After 100th stripping/plating process, a ring XRD pattern can be seen in Li@LiNO_3_ while Li@CA-LiNO_3_ still exhibits polycrystalline crystal structure (Fig. 3e, f). The monotonically increase of Li_2_O and Li_2_CO_3_ coupling with powder metalic Li emergence in Li@LiNO_3_ unveils its severe dead Li formation and side reaction (Supplementary Fig. 16). This could be an indicator of electrode failure of the cell and the penetration of the SEI and dendrite growth towards the cathode. Thus, this multifunctional SEI layer homogenizes Li deposition, manipulates Li’s crystal structure and eliminates its texture formation.

### Morphology characterization of LMA

The surface morphology of LMA was analyzed by SEM and atomic force microscopy (AFM) under the peak force tapping mode. A porous and loose surface layer with large cracks can be observed on Li@LiNO_3_ after 10th stripping/plating process (Fig. 3g and Supplementary Fig. 17A). The microsized Li grain with limited interfaces affects the Li^+^ consumption, which influences the uniformity of the stripping process, leading to the crack formation. The sluggish ion conduction of thick SEI results in preferentially reduced Li^+^ diffusion and Li dendrite sprout on the interface. In contrast, smooth surface with abundant nanosized spherical Li underneath can be observed in Li@CA–LiNO_3_ (Fig. 3h and Supplementary Fig. 17B). This nanostructured LMA enables fast atomic diffusion owing to the less densely packed structure. The intimately contacted nanosized grain provides prominent high-diffusivity paths for expedite conduction. The AFM images also reveal the uneven surface of Li@LiNO_3_ with abundant randomly distributed particles (Fig. 3k and Supplementary Fig. 18A). However, a continuous film with a smooth morphology is observed on Li@CA–LiNO_3_ (Fig. 3l and Supplementary Fig. 18B), indicating its good surface uniformity with inhibited Li dendrite growth. After 100th stripping/plating process, the Li@LiNO_3_ exhibits abundant mossy-like Li dendrite growth and thickened “dead” Li layers on the surface (Fig. 3i and Supplementary Fig. 19A, B) while Li@CA–LiNO_3_ still demonstrates nanosized spherical Li morphology without dead Li or dendrite formation (Fig. 3j and Supplementary Fig. 19C, D). The optical observation of fresh and postmortem Li@LiNO_3_ was further provided to examine its surface morphology variation (Supplementary Fig. 20). Clearly, fresh electrode demonstrates smooth surface while the postmortem electrode undergoes severe electrode pulverization by showing surface deformation and black powdery Li formation. However, the Li@CA–LiNO_3_ under the same condition still exhibits shiny and flat surface, indicating its efficient LMA protection. This well-defined unique nanosized Li deposition with isotropic spherical features ensures the smooth surface formation without any sharp tip, thus avoiding serious safety hazards caused by dendritic Li growth. Besides, spherical Li exhibits minimum surface-to-volume ratio, which signifies higher CE and longer cycle life by exhibiting less side reactions between fresh Li and electrolyte^48^. Mechanical properties are quantitatively described by the Young’s modulus obtained from force indentation curves (Fig. 3n, o). Since the Young’s modulus of Li is highly dependent on the orientation, the strong texture formation of Li metal results in a concentrated Young’s modulus distribution at ~1 GPa. In addition, Li@CA–LiNO_3_ shows a non-normal distribution Young’s modulus, confirming the non-preferred orientation of Li deposit. Li@CA–LiNO_3_ exhibits an average Young’s modulus of 6.61 GPa, which is three times higher than that of Li metal (2.11 GPa). It is reported that the surface layer with a Young’s modulus greater than twice of Li is able to inhibit Li dendrite formation^49,50^. Therefore, this stiff surface of Li@CA–LiNO_3_, which may be related to fine-grain strengthening effect of nanosized Li, is capable of inhibiting dendrite growth. To further understand the formation of Li nanosphere, DFT calculation was provided to gain insights into the interaction between carboxylic group and Li in CA–Li. The computed results show that the BE of Li with different chain lengths are all high enough to demonstrate strong affinity to trap Li (Fig. 3m). The suitable BE of long chain CA–Li reveals its appropriate Li adsorption/desorption process, which favors Li^+^ transportation within the film. The optimized geometrical structure of CA–Li polymers show that Li atom is chemically trapped and intimately contacted with other Li atoms on adjactent monomers owing to its steric repulsion. This confinement is more evident with the polymer chain increasing, which constrains Li atoms together for nucleation/growth and Li nanosphere formation. This result is consistent with GIXD and SEM observations, confirming the regulation of CA–Li on the adsorption and nucleation behavior of Li. Therefore, this multifunctional SEI governs morphological and structural change to deliver isotropic spherical Li nanocrystal, which is capable of facilitating surface diffusion and, favoring homogenized Li^+^ nucleation/deposition to achieve dendrite-free LMA (Supplementary Fig. 21).

### Electrochemical cycling performance

**Fig. 4:**
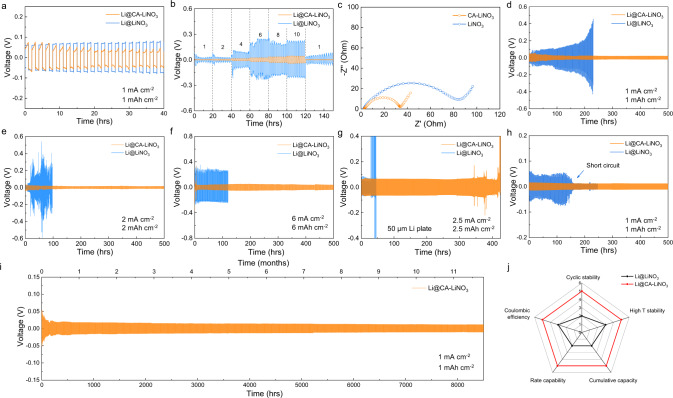
Cycling stability of LMA in Li@CA–LiNO_3_ and Li@LiNO_3_ electrolyte. **a** Stripping/plating profiles. **b** Rate capability under various current densities from 1 to 10 mA cm^−2^. **c** EIS spectra. Time–voltage profiles under **d** 1 mA cm^−2^ with 1 mAh cm^−2^, **e** 2 mA cm^−2^ with 2 mAh cm^−2^ and **f** 6 mA cm^−2^ with 6 mAh cm^−2^. **g** Time–voltage profiles of thin LMA. **h** Time–voltage profile under raised temperature of 60 °C. **i** Long-term cyclic stability. **j** Comparison of performance in terms of rate capability, coulombic efficiency, cyclic stability, cumulative capacity, and high-temperature stability.

The electrochemcial performances were further evaluated in symmetric cells. Figure 4a shows the stripping/plating profiles of LMA in LiNO_3_ and CA–LiNO_3_ electrolyte (noted as Li@LiNO_3_ and Li@CA–LiNO_3_). Better cyclic stability with lower potential hysteresis can be obeserved in Li@CA–LiNO_3_. The rate performance in Fig. 4b indicates that Li@LiNO_3_ succumbs to substantial voltage fluctuation during stripping/plating process, demonstrating its increased polarization. However, Li@CA–LiNO_3_ exhibits a much lower overpotential and flat voltage plateau at all current densities, corresponding to its good SEI stability and desirable Li^+^ conduction. The EIS spectra in Fig. 4c also reveal a much reduced charge transfer resistance (*R*_ct_) of Li@CA–LiNO_3_, indicating its faster Li^+^ transportation in SEI layer. Figure 4d shows the time–voltage profiles under the current density of 1 mA cm^−2^ and capacity of 1 mAh cm^−2^. The Li@LiNO_3_ demonstrates severe polarization after 100th stripping/plating process attributed to the continuous electrolyte decomposition and dead Li formation, while CA–Li@LiNO_3_ exhibits superior cyclic stability without potential fluctutation. The GIXD and SEM observation congruously confirms ever-increasing dead Li formation during cycling. Even under raised current density and capacity of 2 mA cm^−2^/2 mAh cm^−2^ and 6 mA cm^−2^/6 mAh cm^−2^, Li@CA–LiNO_3_ still demonstrates a remarkable cyclic stability (Fig. 4e, f). The practical application of high-energy-density LMB requires the LMA with thin thickness (≤50 μm). As such, the cycling performance of 50 μm Li plate was evaluated (Fig. 4g). Li@CA–LiNO_3_ exhibits stable stripping/plating process over 150 cycles, which outperforms Li@LiNO_3_, indicating the superior SEI stability for practical application under increased depth of discharge. To investigate its high-temperature stability, cycling performance, and EIS spectra under high-temperature condition of 60 °C was further examined (Fig. 4h and Supplementary Fig. 22). Li@CA–LiNO_3_ delivers a much longer cyclic stability and smaller *R*_ct_ than those of Li@LiNO_3_, indicating the excellent LMA protection under extreme temperature condition. Owing to the SEI structural superiorities, a ultralong-term cyclic stability over 8500 h is achieved in Li@CA–LiNO_3_, corresponding to a superb culmulative capacity of 4.25 Ah cm^−2^, as shown in Fig. 4i. To confirm the improved overall performance raised by multifunctional SEI engineering strategy, the perfromance comparisons are depicted in the radar chart (Fig. 4j). The Li@CA–LiNO_3_ delivers riverting multifunctionalities including remarkable rate capability, CE, cyclic stability as well as high cumulative capacity and high-temperature stability, which are far beyond than those of Li@LiNO_3_. These significantly improved perfromances are much competitive comparing with other LMA reported from literatures (Supplementary Table 2).

The efficient LMA protection capability of CA–Li is prominent when compared with other artificial SEI layer. For comparison, PVDF-coated LMA, the commonly used polymeric layer for LMA protection, was synthesized as reported from literature^51^. The PVDF-coated LMA exhibits a stable platting/stripping process in the initial cycling stage under 1 mA cm^−2^/1 mAh cm^−2^ (Supplementary Fig. 23). However, this anode experiences large polarizations after 150 h cycling, which can be attributed to the non-uniform Li deposition and growth of lithium dendrite. The morphology evolution of PVDF-coated LMA cycled in LiNO_3_ electrolyte were further evaluated. A uniform polyhedron Li deposition can be obtained after the first plating process (Supplementary Fig. 24). However, a cracked PVDF film with agglomerated Li particle underneath can be observed after 10th stripping/plating process, indicating that a non-uniform Li nucleation/deposition process. After 100th stripping/plating process, the large bulk-type Li can be observed while the initially uniform PVDF protection layer now cannot be found, which could be related to the breakage and pilling off of the PVDF layer. The severe Li particle aggregation and non-uniform Li deposition can be ascribed to the slow diffusion and fast consumption of Li^+^ upon deposition and the rapid decline of ion concentration in the SEI layer. The slowly diffused Li^+^ in the SEI layer induced inhomogeneous Li plating and the dendritic Li growth under the diffusion‐controlled condition^52^. The BE of PVDF with Li was further calculated, which shows very low adsorbability on Li, rendering an unstable film formed on the surface of Li and limited capability to homogenize Li deposition and inhibit dendrite growth (Supplementary Fig. 25). In contrast, the LC exhibits stronger chemical interaction with Li, which offers potent binding capability to cover the surface of Li, leading to stable operation over long-term cycling.

**Fig. 5:**
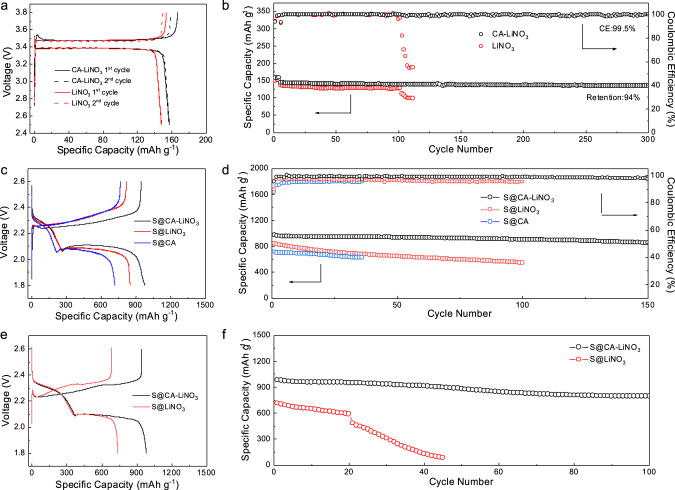
Electrochemical performance under practically relevant conditions. Li–LFP full-cell performance under high LFP loading of ~18 mg cm^−2^, low N/P ratio of ~2 and lean electrolyte content of 6 g Ah^−1^: **a** GCD profiles at 0.2 C and **b** cyclic performance at 1 C. Li–S electrochemical performance of S@CA–LiNO_3_, S@LiNO_3_, and S@CA: **c** GCD profiles and **d** cyclic performance under the discharge current density of 0.1 C under raised sulfur loading of ~10 mg cm^−2^ and low E/S ratio of 4.5 ml g^−1^. Li–S full-cell performance: **e** GCD profiles and **f** cycling performance under low N/P ratio of ~1.5 and lean electrolyte content of 6 g Ah^−1^.

LFP cathode with a practical mass loading of ~18 mg cm^−2^ and lean electrolyte content of 6 g Ah^−1^ was employed to analyze the electrochemical peformance for LMB practical application (Supplementary Fig. 26). The galvanostatic charge–discharge (GCD) profiles of Li–LFP performance with CA–LiNO_3_ electrolyte (LFP@CA–LiNO_3_) exhibit a higher discharge capacity and similar charge–discharge plateaus compared with Li–LFP battery in LiNO_3_ electrolyte (LFP@LiNO_3_). The CV curve of LFP@CA–LiNO_3_ exhibits a relatively large potential hysteresis during intital anodic and cathodic scanning and similar voltage difference in the second scanning compared with LFP@LiNO_3_. After activation, the EIS spectra of LFP@CA–LiNO_3_ show a much lower *R*_ct_ than LFP@LiNO_3_, confirming its fast charge transfer kinetics. The cyclic performance of LFP@LiNO_3_ demonstrates a rapid CE drop after 150 cycles, which is related to fast electrolyte drying. However, the utilization of Li during cycling is remarkably promoted in LFP@CA–LiNO_3_ with a high CE over 99.5% after 300 cycles. The cells after 100 cycles were disassembled and the morphology of separator after cycling was evaluated. Notably, the LFP@CA–LiNO_3_ separator is still wet with abundant electrolyte inside while the LFP@LiNO_3_ separator is almost dried (Supplementary Fig. 27). Li–LFP full-cells under low N/P ratio of ~2 were further probed, as shown in Fig. 5a, b. Clearly, LFP@CA–LiNO_3_ still displays a higher discharge capacity and similar voltage difference when compared with LFP@LiNO_3_, indicating their similar kinetics. A significant drop of capacity and CE can be observed in the 102th cycles of LFP@LiNO_3_. However, LFP@CA–LiNO_3_ delivers a decent cycling performance upon 300 cycles. The cycling performance was further evaluated under high-temperature condition (Supplementary Fig. 28). A higher discharge capacity and prolonged cycle life can be observed in LFP@CA–LiNO_3_, confirming its superior LMA protection capabilities under practical harsh condition.

The electrochemcial performances of CA–LiNO_3_ electrolyte were further evaluated in Li–S batteries to reveal the SEI stability for polysulfide (PS) corrosion inhibition and sulfur kinetics promotion by CA. The Li–S batteries were assembled using CA, LiNO_3_, and CA–LiNO_3_ electrolyte (noted as S@CA, S@LiNO_3_, and S@CA–LiNO_3_), separately. The introduction of CA additive faciliates the short chain LPS (Li_2_S/Li_2_S_2_) dissociation, which reduces the formation of insoluble Li_2_S particles and improves the reversible and sustainable Li–S redox reaction. CV, GCD profiles, and EIS spectra were used to identify their redox reactions process. S@CA–LiNO_3_ exhibits the typical two discharge plateaus around 2.35 and 2.1 V, corresponding to the reduction of sulfur into long chain LPS and then transformation to insoluble Li_2_S_2_ and Li_2_S (Supplementary Fig. 29A)^53^. Besides, the charge plateau at 2.3 V is related to the oxidation of Li_2_S to elemental sulfur. Although S@CA exhibits improved Li–S kinetics, it delivers the lowest discharge capacity and much lower CE. In addition, the S@CA–LiNO_3_ keeps the lowest potential hysteresis and highest discharge capacity of 1141.5 mAh g^−1^ compared with S@LiNO_3_ and S@CA. The absence of LiNO_3_ results in insufficient inorganic Li formation in SEI layer, rendering undesirable side reaction between LPS and Li. The limited oxynitride inside SEI results in low ionic conductivity, rendering sluggish redox reaction and high potential hysteresis in S@CA. The CV curves of S@CA–LiNO_3_ demonstrate the most pronouced reduction and oxidation peaks with lowest potential hysteresis, consistent with the GCD results (Supplementary Fig. 29B). The EIS spectra of S@CA–LiNO_3_ hold the smallest *R*_ct_ (Supplementary Fig. 29C), indicating its best charge transportation in the Li–S cell^54^. Galvanostatic intermittent titration techniques (GITT) were also performed (Supplementary Fig. 30) to check the electrochemical equilibria during discharge/charge process. S@LiNO_3_ shows another long discharge plateau below 1.8 V, which corresponds to the LiNO_3_ decomposition. S@CA–LiNO_3_ exhibits no LiNO_3_ decomposition below 1.8 V, indicating the reduced LiNO_3_ reduction. A much lower overpotential can also be observed in the intial charge stage, corresponding to its favored Li_2_S oxidation process. Moreover, S@CA–LiNO_3_ manifests the best rate capability and cyclic stability (Supplementary Fig. 29D and E). By virtue of the stable SEI and favorable kinetics, a remarkable Li–S performance is achieved, leading to a superior redox reaction kinetics and prolonged life span. The LMA protection capability is further revealed from SEM and optical observations of LMA after 500 cycles (Supplementary Fig. 31). The LMA of S@CA–LiNO_3_ still keeps its shiny and uniform surface while the anode of S@LiNO_3_ shows dark color from optical image and abundant microcracks under SEM investigations, corresponding to its severe corrosion. To verify that S@CA–LiNO_3_ is practically feasible for the battery design and meets its high-energy-density requirement, the electrochemical performance under high sulfur loading of ~10 mg cm^−2^ and limited E/S ratio of 4.5 mL g^−1^ was investigated, as shown in Fig. 5c, d. Notably, S@CA–LiNO_3_ manifests the highest areal capacity, lowest polarization and best cyclic stability over 150 cycles with a capacity retention over 80%. Full-cell performance was further examined at a very low N/P ratio of ~1.5 and limited electrolyte content of 6 g Ah^−1^. Compared with S@LiNO_3_, S@CA–LiNO_3_ shows a longer second discharge plateau with a higher initial discharge capacity and lower potential hysteresis, indicating its fast Li–S kinetics (Fig. 5e, f). The much improved cyclic stability of S@CA–LiNO_3_ under low N/P ratio indicates its limited side reaction and electrolyte consumption of LMA. Therefore, the introduction of CA not only facilities stable SEI formation in S@CA–LiNO_3_ and prevents LMA from side reaction and PS corrosion, but also regulates Li nucleation/deposition process and suppresses Li dendrite growth, leading to enhanced redox reaction kinetics and much improved cycle life in high-energy-density LMB.

## Discussion

The strong adhesion of marine mussels to versatile solid surface under saline water inspired us to design catechols family molecules as additives to construct stable SEI. The versatilities of catechols are capable of being functionalized by grafting different functional group on the backbone. For example, the acrylic group, of which the polymerization can be initiated by Li metal, is able to be grafted and endows the molecule with capability of polymerization. Design a catechol molecules with multiple functional groups offers a new interfacial engineering strategy to construct multifunctional SEI layer. As proof of concept, we firstly introduce CA as electrolyte additive to enable uniform polymeric film formation adapting to its surface morphology via in situ polymerization. The strong surface adsorption followed by anionic polymerization of CA ensures the thin film CA–Li formation on Li metal, while the inorganic Li compound is further inlaid inside film during electrochemical reduction process, leading to the multifunctional SEI formation.

A spherical Li deposit with non-preferred orientation and small crystal size is capable of achieving dendrite-free Li anode. The subtle control of Li^+^ nucleation under SEI layer is crucial in generating uniform spherical Li deposits. In particular, the structure/morphology evolution of LMA is visualized and the dendrite suppression mechanism is revealed. In the presence of CA, the surface of LMA gradually changes from textured microsized Li to polycrystalline grain with random orientation. DFT calculations indicate that Li^+^ are prone to be adsorbed on CA–Li layer due to the suitable binding interactions. The multiple functional group of CA–Li offers steric confinement to constrain Li nucleation/growth process and even the distribution of Li^+^ to remediate the locally enhanced Li^+^ flux. As a result, this multifunctional SEI layer regulates the morphology, microstructure and growth orientation of Li deposit over the course of electrochemical process, which endows the Li with spherical morphology, nanosized crystals and limited dead Li formation. Moreover, the high Young’s modulus of Li@CA–LiNO_3_ is able to conform to the mechanical deformation, indicating another possible reason for suppressing Li dendrites.

In conclusion, a novel and straightforward strategy has been developed to construct hybrid multifunctional SEI by using CA as electrolyte additive toward dendrite-free, long lifespan LMB application. The as-developed Li@CA–LiNO_3_ exhibits high cumulative capacity, superb rate performance, and ultra-long cyclic stability, which far outperform Li@LiNO_3_ under both practical operation condition and extreme temperature applications. Attributed to these structural superiorities, the Li–S and Li–LFP full cells deliver impressive cycling performances under practical relevant N/P ratio and lean electrolyte content. This facile strategy by directly introducing catechol family molecules into electrolyte advances and provides a new direction on electrolyte design for related energy storage systems, which can also be transferred to the battery industry. The synergistic effect by using catechol family molecules and other additives in LMB should also be evaluated to explore the performance enhancement and promote its practical applications.

## Methods

### Electrolyte preparation

CA was purchased from Sigma-Aldrich. Li metal was purchased from China Energy Lithium Co., LTD. The conventional DOL/DME electrolyte contains 1 M LiTFSI in DOL/DME solution (v/v = 1:1). The 1% CA electrolyte was prepared by adding 1 wt% CA in conventional electrolyte and stirring for 24 h. The CA–LiNO_3_ electrolyte was prepared by adding 0.1–1 wt% CA and 2 wt% LiNO_3_ in conventional electrolyte and stirring for 24 h. The LiNO_3_ electrolyte was prepared by adding 2 wt% LiNO_3_ in conventional electrolyte and stirring for 24 h.

### Symmetrical cell fabrication

In Li–Li symmetric cell, the Li plates were used as working and counter electrode. The electrochemical performance test was carried out in CR2032-type coin cell by using lithium plate electrode, electrolyte, and polypropylene separators (Celgard 2500) in an Ar-filled glove box with a water and oxygen level below 0.5 ppm. The pristine Li plates with 15.8 mm diameter was used with a thickness of ~450 μm. The amount of electrolyte used was 30 μL. After assembling, the battery was used for electrochemical testing after 12 h rest.

### Materials characterization

AFM (Asylum Research Cypher S AFM) and AC160TS-R3 tip (Olympus) was used to analyze the surface smoothness and Young’s modulus. Sneddon model was used to fit the force curves. Over 100 spots were randomly chosen on the selected region and the number of Young’s modulus in the specific range were counted. AFM force curves were conducted in glove box to avoid sample oxidation. X-ray photoelectron spectroscopy was used to analyze the surface components and chemical composition. SEM (Hitachi Regulus 8230+Oxford Ultim Extreme) was used to examine the surface morphology. All the cycled electrodes were washed by DME for three times to remove residual lithium salt, vacuum dried in glovebox and sealed in Ar-filled box for sample transfer. Fourier transform infrared spectrometry (FTIR, NICOLETIN10) was used to obtain the FTIR and ATR spectra. The ^1^H and ^7^Li NMR experiments were performed on a Bruker 300 MHz spectrometer. The deuterated dimethylsulfoxide (DMSO) was employed as the dispersion. The total volume of the solution in NMR test is 1 mL. UV–vis spectra were collected by Thermal Scientific GENESYS 10S spectrophotometer.

### Synchrotron 2D GIXD measurement

The Synchrotron 2D GIXD were performed on VESPERS beamline at the Canadian Light Sources. The LMA after cycling were taken out from coin cell and carefully washed for three times in the Ar-filled glovebox. The Li sample was further mounted in the Kapton film covered sample mount for experiment to avoid air exposure. The beam size used for experiment is ~3 × 6 μm. The incident angle is 0.2°. The energy of X-ray beam used for GIXD is 8 keV. The beam diffracted from Li plate was collected by a 2D area detector centered at 40° and located 120 mm away from the sample, which covers a 2*θ* angular range of 10–70°. The XRD 2*θ* plot is integrated from 2D pattern processed by XMAS software.

### Electrochemical testing

A LAND CT2001 electrochemical testing system was used to measure the electrochemical performance of these cells. The CV and EIS measurements were carried out on a Biologic multi-channel electrochemical workstation over the frequency from 100 mHz to 100 kHz with an amplitude of 10 mV. The CV measurement of other samples were measured by Gamry potentiostat. For activation, the cell was rested for 12 h and pre-cycled for 10 cycles. A cut-off voltage of 1 V was applied in symmetrical cell and Li–Cu half-cell test.

## Supplementary information

Supplementary Information

## Data Availability

All data needed to evaluate the conclusions in the paper are present in the paper and/or the Supplementary Materials. Additional data related to this paper may be requested from the authors.
