# Supplementary material for: Constructing multifunctional solid electrolyte interface via in-situ polymerization for dendrite-free and low N/P ratio lithium metal batteries
Source: Nat Commun. 2021 Jan 8;12:186. doi: 10.1038/s41467-020-20339-1 (PMC7794354; doi:10.1038/s41467-020-20339-1)
Supplement: Supplementary file 1 — Supplementary Information [file 41467_2020_20339_MOESM1_ESM.pdf]

**Supporting Information**

# Construct Multifunctional Solid Electrolyte Interface *via In-Situ* Polymerization for Dendrite-Free and Low N/P Ratio Lithium Metal Batteries

Luo *et al.*

## Supplementary Methods

### Experimental Section

**Synthesis of sulfur cathode materials:** To obtain sulfur electrode, sulfur powder was mixed with porous carbon (KJ600) with mass ratio of 4:1, well grinded in mortar and heated under 155°C for 12 hrs. The obtained material was mixed with polyvinylidene fluoride (PVDF), super P and were dispersed in N-methyl-2-pyrrolidone (NMP). The mass ratio of active material: Super P: PVDF is 8:1:1. The raw materials were mixed uniformly to obtain a slurry and coated on aluminum foil. The electrode was further dried under 70°C for 12 hrs and punched to disks with 15 mm in diameter. For high sulfur loading experiment, the porous carbon fiber paper was used as sulfur host.

**LFP cathode materials:** The LFP powder, super P and PVDF were well grinded in NMP solution with a mass ratio of 90:5:5 and casted on carbon coated aluminum foil. The electrode was further dried under 70°C in vacuum oven for 24 hrs and punched to disks with 15 mm in diameter. The average areal LFP loading was ~18 mg $cm^{-2}$. The LFP cathode with an areal capacity of 3 mAh $cm^{-2}$ was employed for Li-LFP full cell. Li metal with a thickness of 30 μ m was used as anode, corresponding to an areal capacity of 6 mAh $cm^{-2}$. Therefore, the N/P ratio can be determined as ~2.

**Computational methods:** Density functional theory (DFT) calculations was employed to investigate the reduction processes of the CA additive. The specific structures were fully optimized by the B3PW91 method with the basis set of 6-311++G (d, p). The polarized continuum model (PCM) as implemented in Gaussian09 was used to describe the implicit solvent effect on the reduction process. The binding energy calculations were performed via the Vienna ab initio simulation package (VASP) with Perdew-Burke-Ernzerhof (PBE)

to calculate electron exchange-correlation interactions. The adsorption energies with different chain length of CA-Li ($E_n$) was determined by the following expression:

$$E_n = (E_{total} - E_{polymer} - E_{Li}) / \text{number of monomers (n)}$$

where $E_{total}$ is the total energy of the adsorbed systems, $E_{polymer}$ is the energy of anion polymer, $E_{Li}$ is the energy of Li.

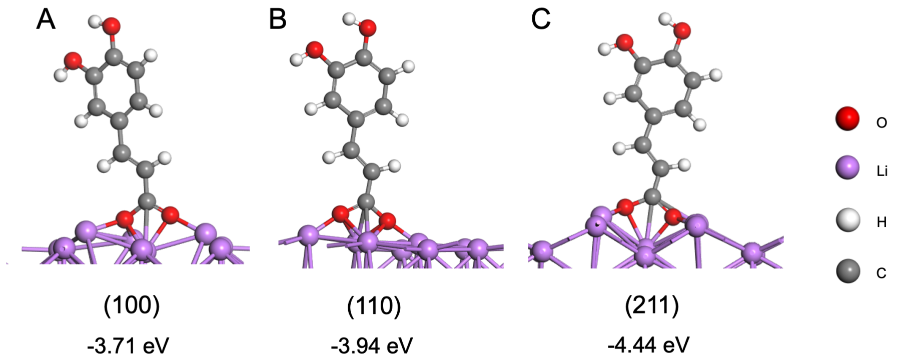


**Supplementary Figure 1.** Geometrical configuration and adsorption energies of CA on Li with different crystalline plane.

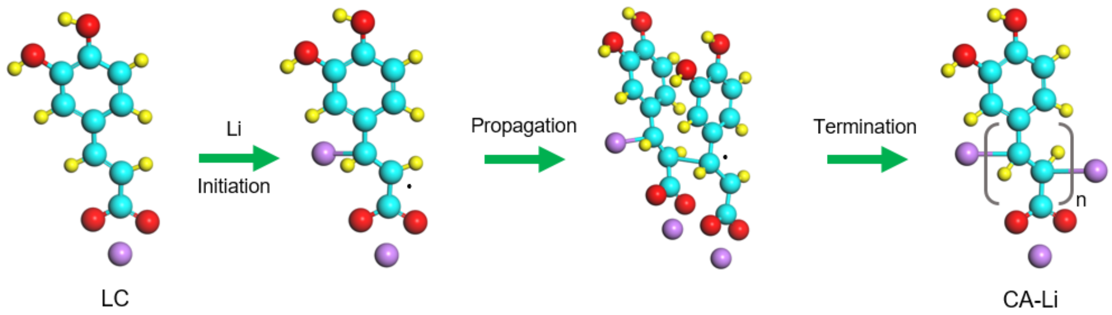


**Supplementary Figure 2.** Schematic illustration of possible anionic polymerization initialized by Li.

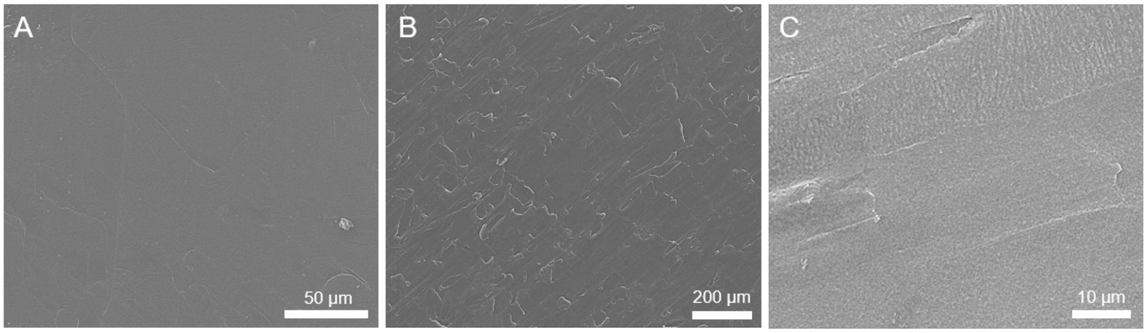


**Supplementary Figure 3.** SEM images of (A) Pristine Li, (B) after dipping 30 μL CA-$LiNO_3$ electrolyte, (C)After 7 days immersion in CA-$LiNO_3$ electrolyte.

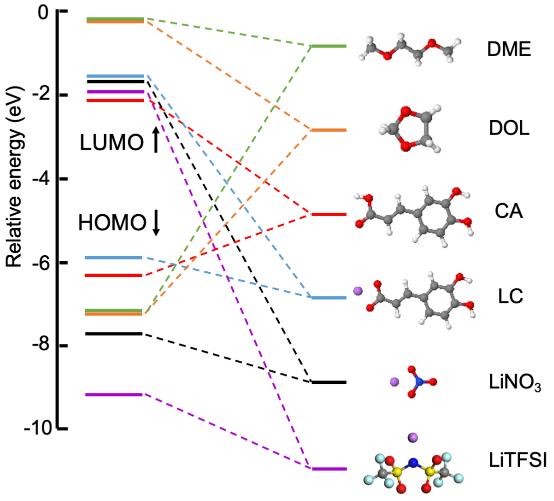


**Supplementary Figure 4.** Diagram of calculated HOMO/LUMO energies of solvent and electrolyte additives.

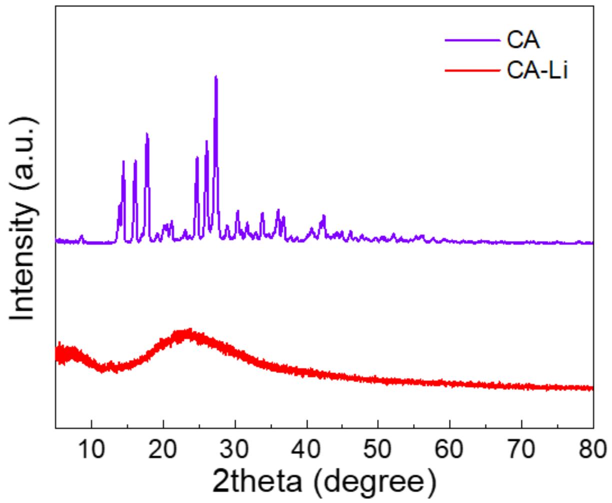


**Supplementary Figure 5.** XRD spectra of CA and CA-Li.

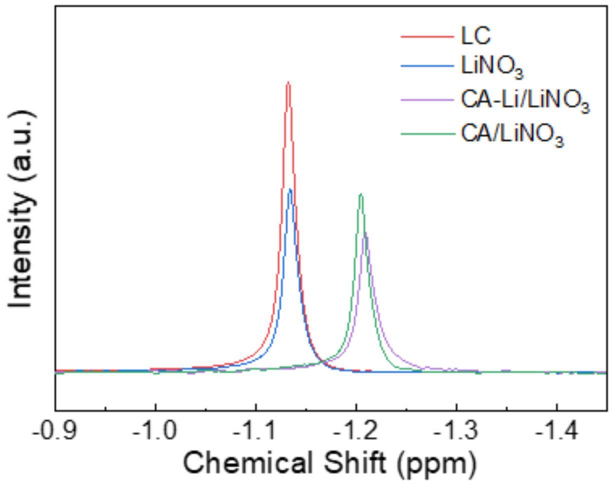


**Supplementary Figure 6.** $^{7}$Li NMR spectra of LC, $LiNO_3$, CA/$LiNO_3$ and CA-Li/$LiNO_3$.

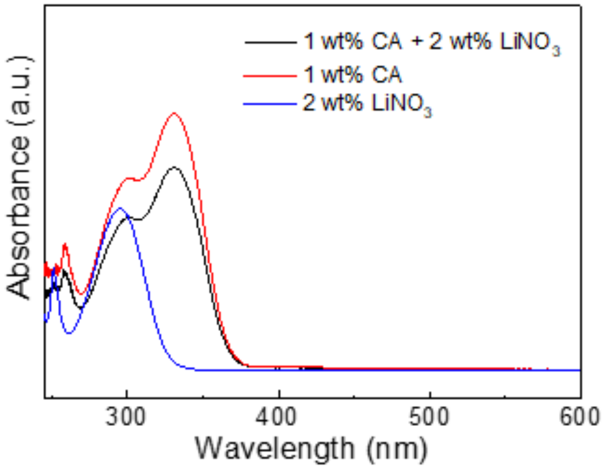


**Supplementary Figure 7.** UV-vis spectra of 1 wt.% CA + 2 wt.% $LiNO_3$, 1 wt.% CA, and 2 wt.% $LiNO_3$.

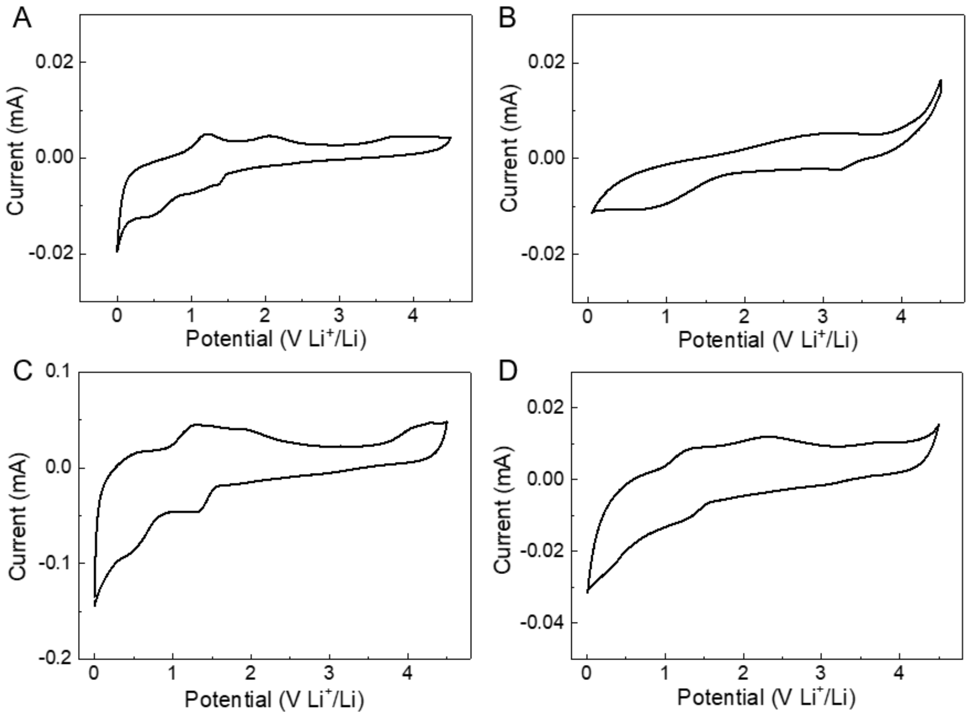


**Supplementary Figure 8.** CV curves of (A) DOL/DME solution, (B) 1% CA DOL/DME solution, (C) conventional electrolyte (containing 1M LiTFSI in DOL/DME solution) and (D) CA electrolyte (containing 1M LiTFSI and 1% CA in DOL/DME solution) under a scan rate of 1 mV $s^{-1}$.

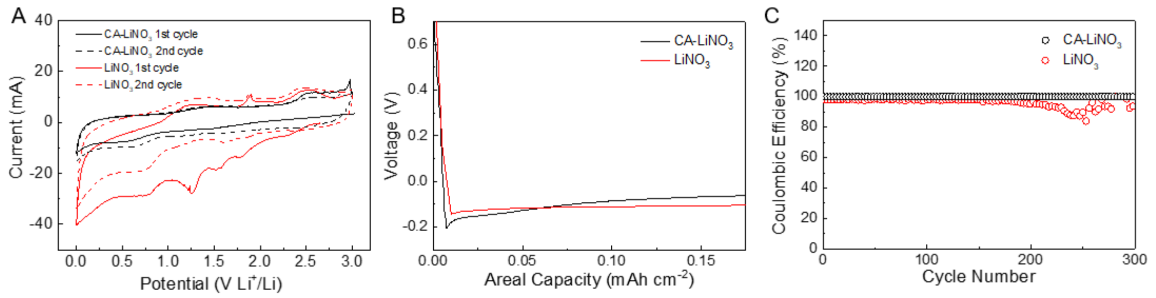


**Supplementary Figure 9.** Electrochemical behavior of Li-Cu half-cell tested in CA-$LiNO_3$ and $LiNO_3$ electrolytes: (A) CV curve with a scan rate of 1 mV $s^{-1}$. (B) Galvanostatic voltage profiles of Li platting on a working Cu electrode at a current density of 1 mA $cm^{-2}$. (C) CE of Li stripping/platting at current density of 1 mA $cm^{-2}$ and capacity of 1 mAh $cm^{-2}$ after 10 cycle's activation.

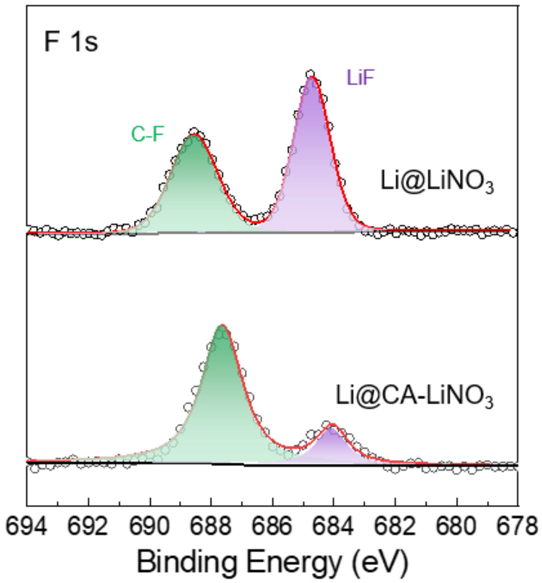


**Supplementary Figure 10.** F 1s XPS spectra of Li@$LiNO_3$ and Li@CA-$LiNO_3$.

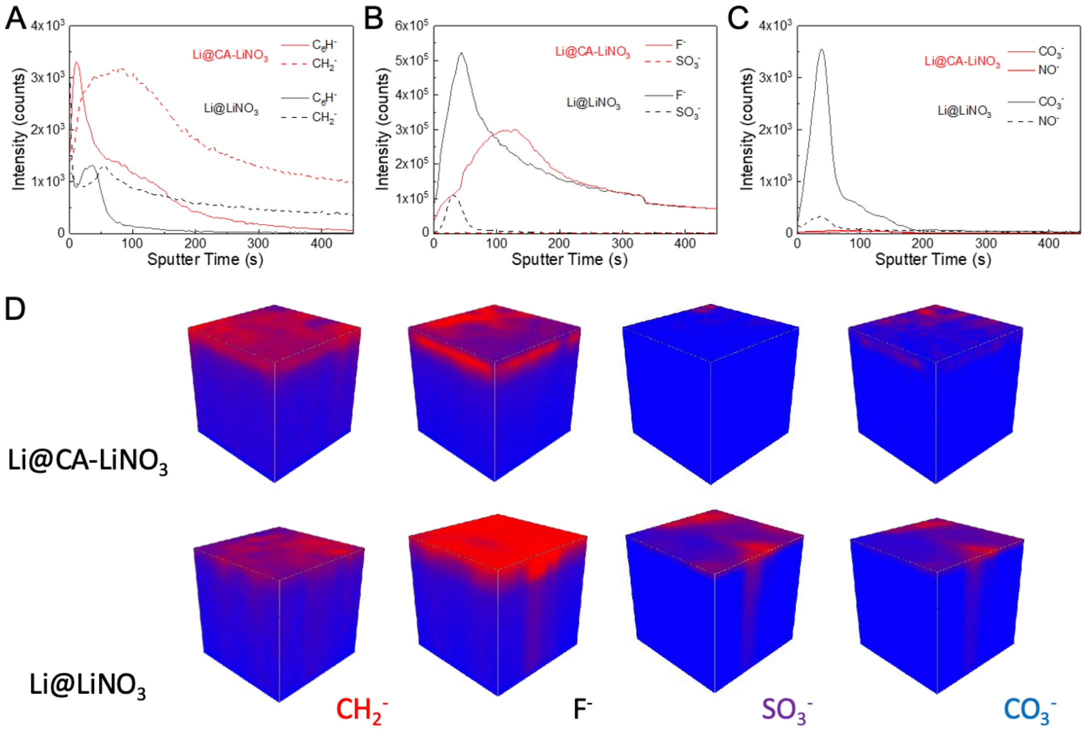


**Supplementary Figure 11.** (A-C) TOF-SIMS depth profiles of Li@CA-$LiNO_3$ and Li@$LiNO_3$ and their (D) 3D reconstructed sputtering images.

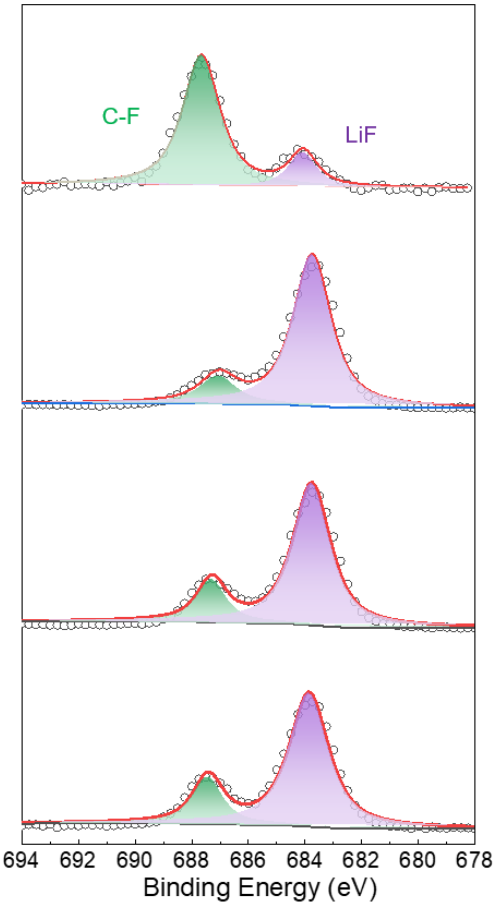


**Supplementary Figure 12.** F 1s XPS depth profiles of Li@CA-$LiNO_3$.

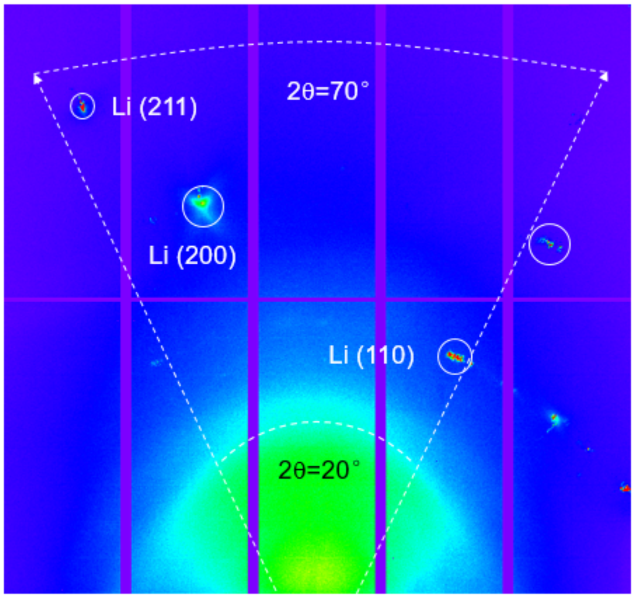


**Supplementary Figure 13.** XRD pattern of pristine Li.

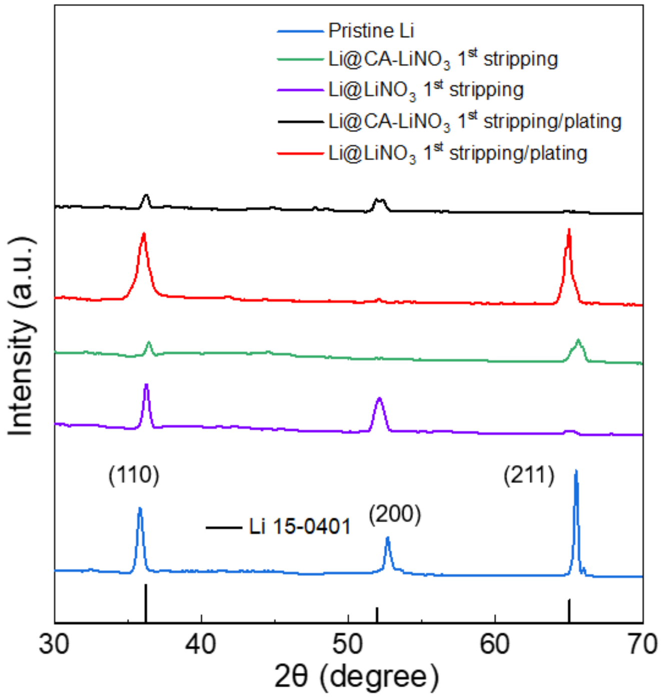


**Supplementary Figure 14.** Integral XRD pattern of pristine Li, Li@$LiNO_3$ and Li@CA-$LiNO_3$ after 1st stripping process and 1st stripping/plating process.

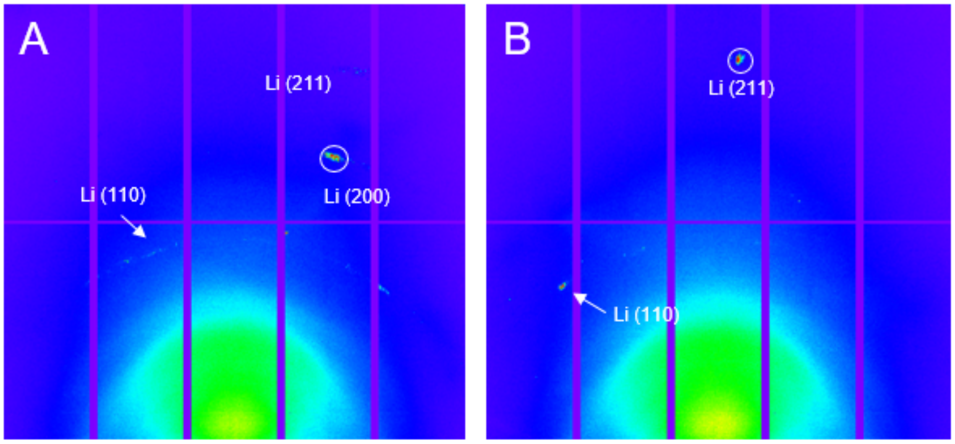


**Supplementary Figure 15.** GIXD pattern of (A) Li@$LiNO_3$ and (B) Li@CA-$LiNO_3$ after 1st stripping process under the current density of 1 mA $cm^{-2}$ and capacity of 1 mAh $cm^{-2}$.

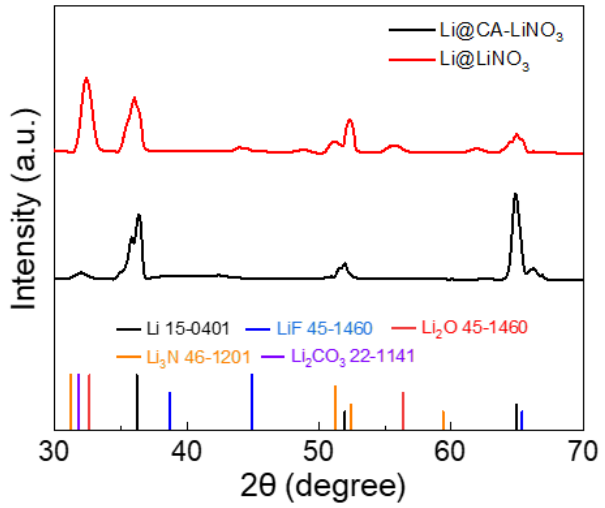


**Supplementary Figure 16.** Integral XRD pattern of Li@$LiNO_3$ and Li@CA-$LiNO_3$ after 100$^{th}$ stripping/plating process.

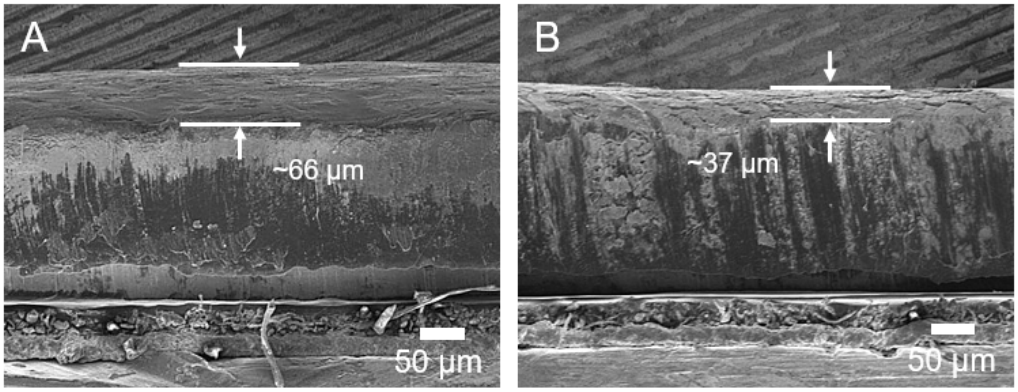


**Supplementary Figure 17.** SEM cross-section image of (A) Li@$LiNO_3$ and (B) Li@CA-$LiNO_3$ after $10^{th}$ stripping/plating process.

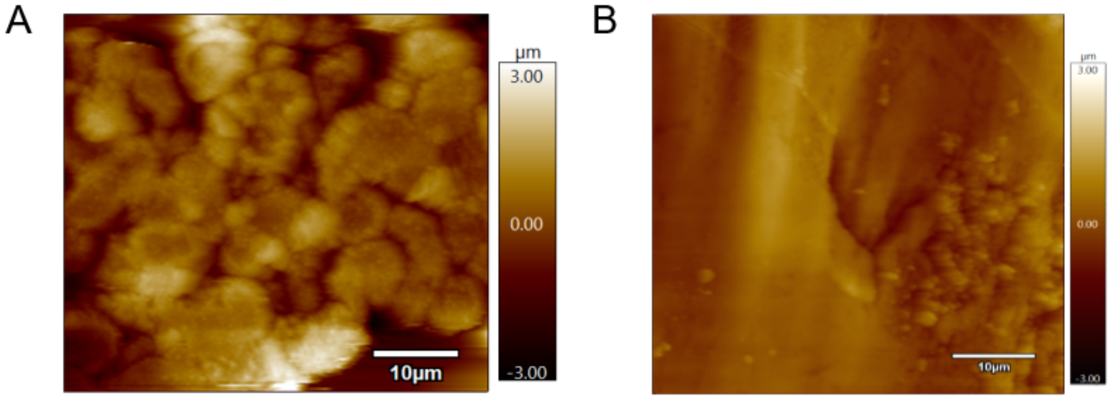


**Supplementary Figure 18.** AFM topography images of (A) Li@$LiNO_3$ and (B) Li@CA-$LiNO_3$ after $10^{th}$ stripping/plating under the current density of 1 mA $cm^{-2}$ with a capacity of 1 mAh $cm^{-2}$.

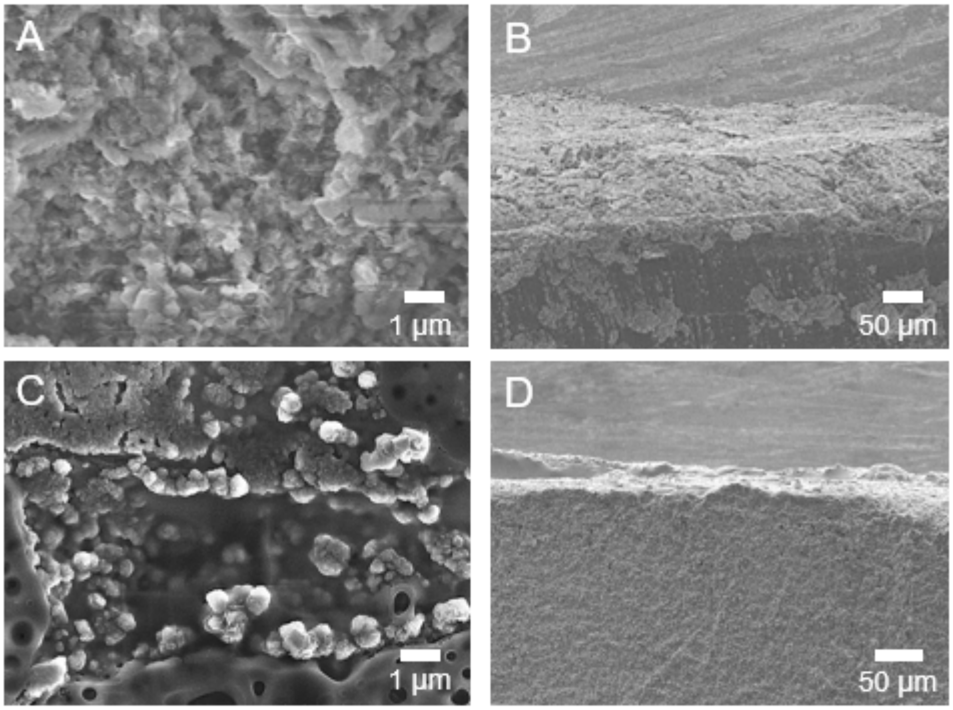


**Supplementary Figure 19.** SEM morphology and cross-sectional images of (A, B) Li@ $LiNO_3$ and (C, D) Li@ CA-$LiNO_3$ after $100^{th}$ stripping/plating process under the current density of 1 mA $cm^{-2}$ and capacity of 1 mAh $cm^{-2}$.

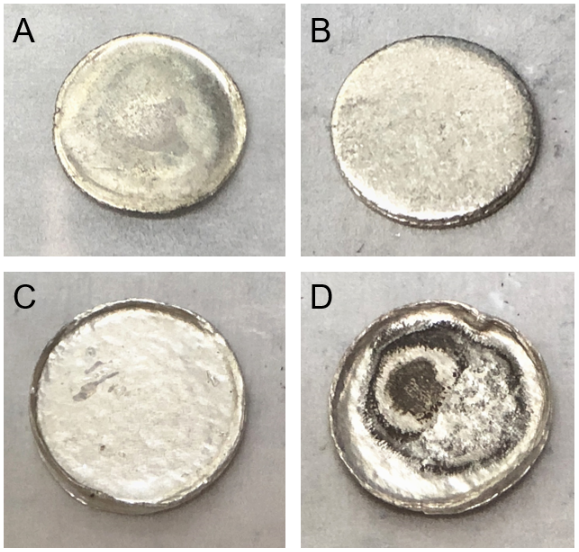


**Supplementary Figure 20.** Optical images of pristine electrode and after cycling of (A, C) Li@CA-$LiNO_3$ and (B, D) Li@$LiNO_3$.

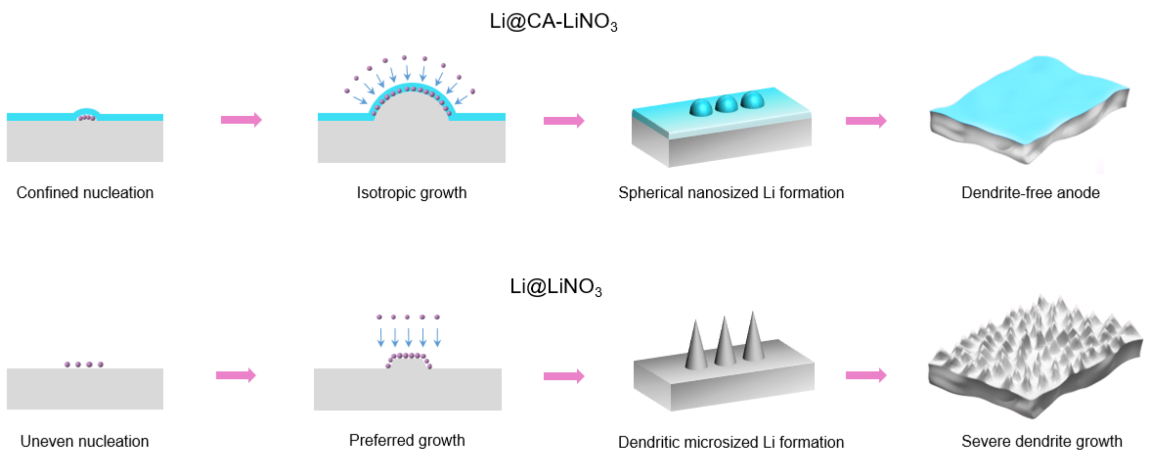


**Supplementary Figure 21.** Scheme illustration of the structure/morphology regulation and dendrite suppression mechanism in Li@CA-$LiNO_3$.

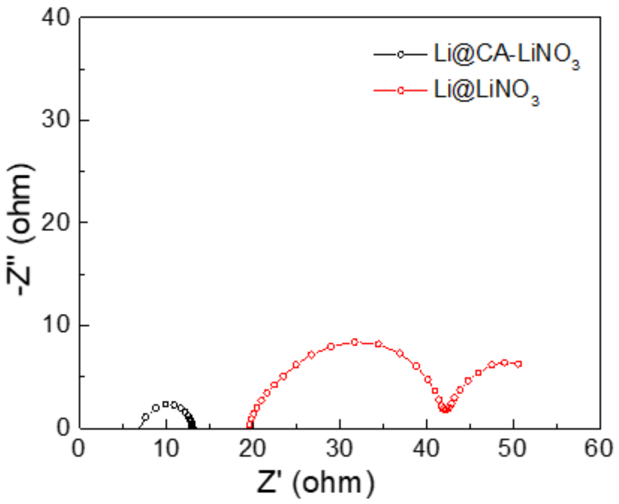


**Supplementary Figure 22.** EIS spectra under 60°C operation condition.

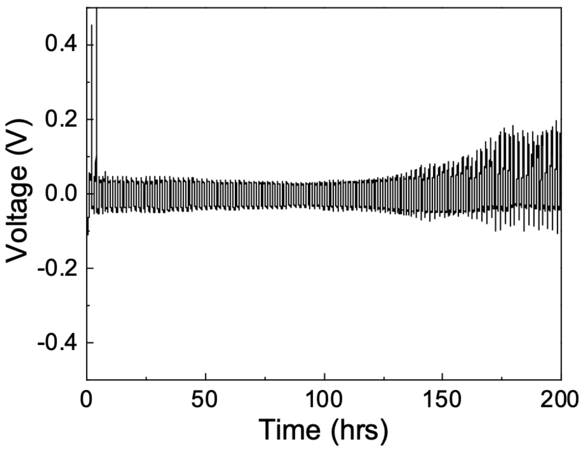


**Supplementary Figure 23.** cycling performance of PVDF coated LMA under the current density of 1 mA $cm^{-2}$ with a capacity of 1 mAh $cm^{-2}$.

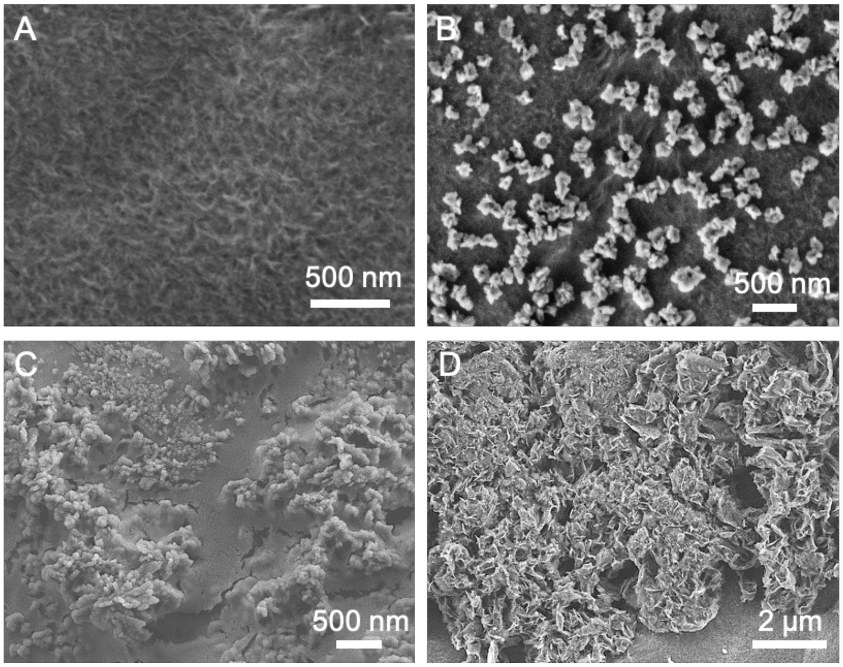


**Supplementary Figure 24.** SEM morphology of (A) PVDF coated Cu foil and LMA cycled in $LiNO_3$ electrolyte after (B) 1$^{st}$ cycle, (C) 10$^{th}$ cycle and (D) 100$^{th}$ cycle.

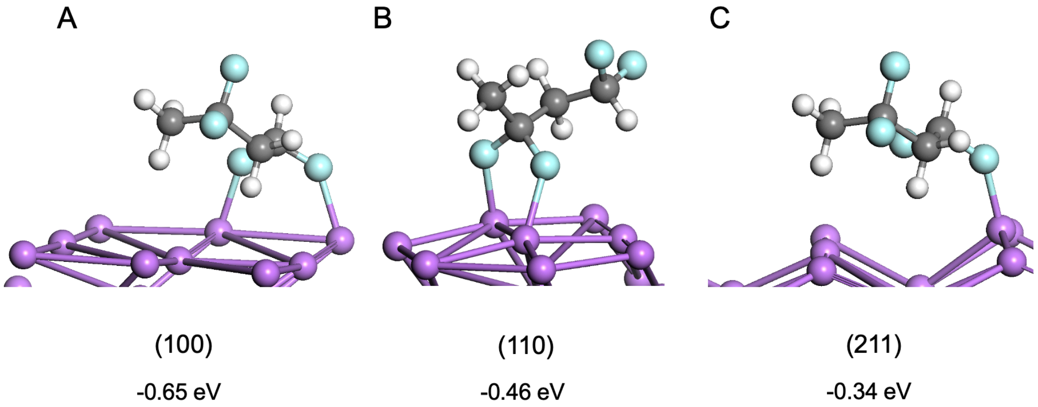


**Supplementary Figure 25.** geometrical configuration and adsorption energies of PVDF on Li with different crystalline plane.

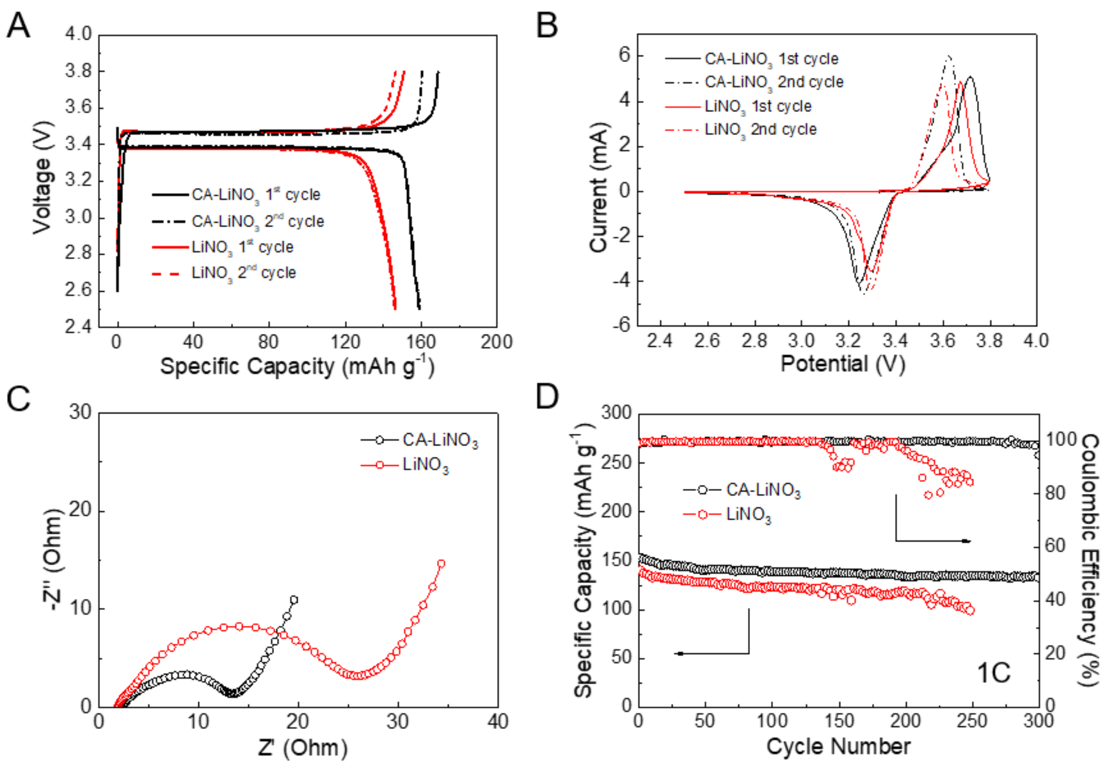


**Supplementary Figure 26.** Li-LFP electrochemical performance of LFP@CA-LiNO3 and LFP@LiNO3. (A) GCD profiles at 0.2 C, (B) CV curves with a scan rate of 1mV $s^{-1}$. (C) EIS spectra and (D) cyclic performance under the current density of 1 C after activation. The cut-off voltage was set as 3.8 V to reduce oxidative polymerization of CA on the cathode.

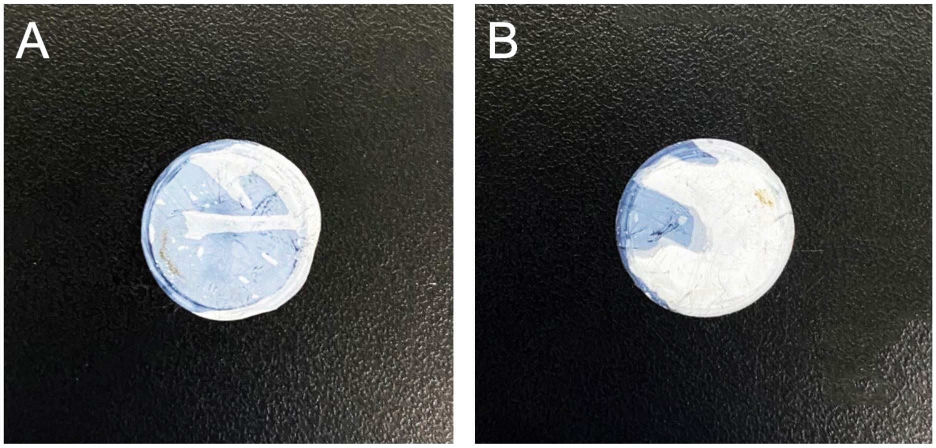


**Supplementary Figure 27.** optical observation of separator taken from disassembled Li-LFP cells after 100th cycles in (A) CA-$LiNO_3$ electrolyte and (B) $LiNO_3$ electrolyte.

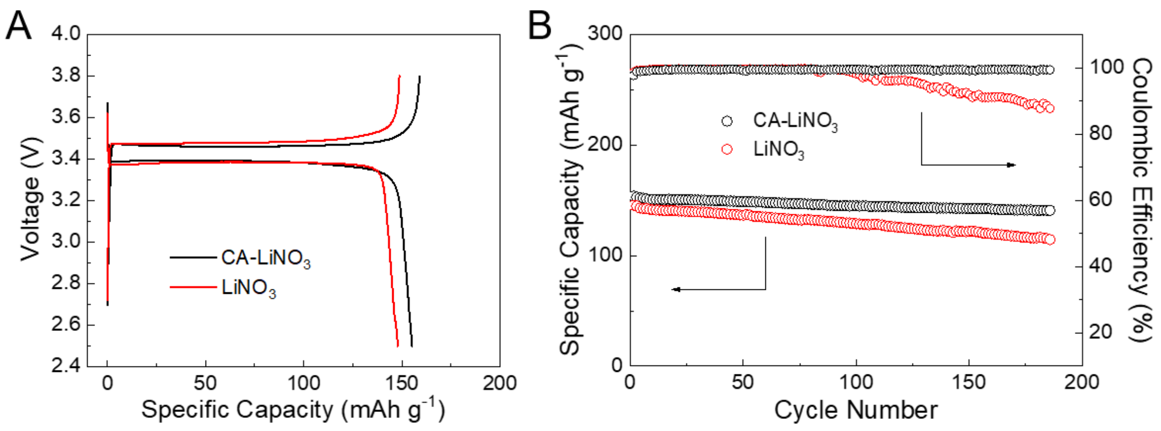


**Supplementary Figure 28.** (A) GCD profiles and (B) cycling performance under the current density of 1 C and 60°C operation condition after activation.

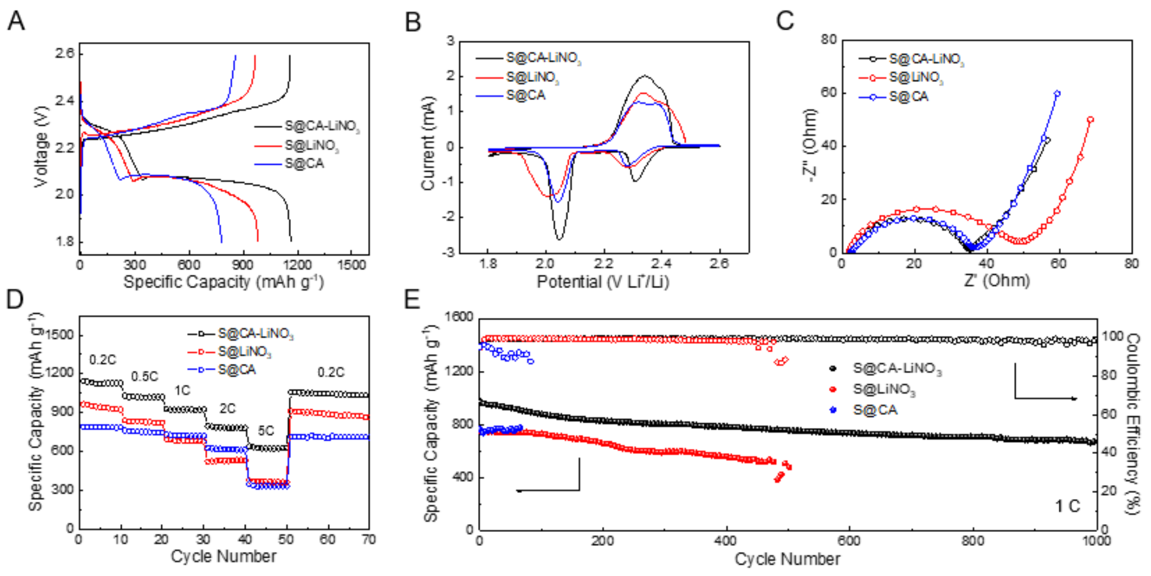


**Supplementary Figure 29.** Electrochemical performance of S@CA-LiNO$_3$ and S@LiNO$_3$: (A) GCD profiles at 0.2 C, (B) CV curves with a scan rate of 1 mV s$^{-1}$, (C) EIS spectra, (D) rate performance and (E) cyclic performance under the current density of 1 C.

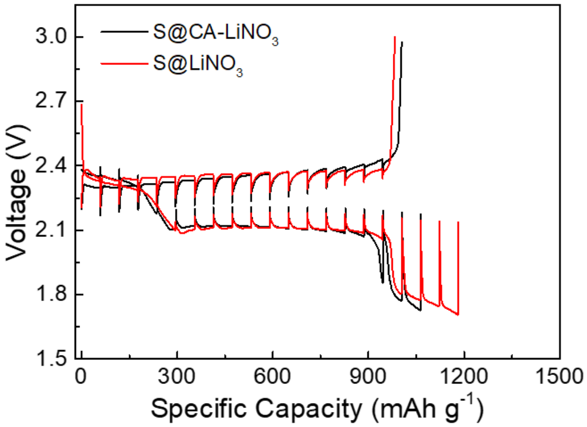


**Supplementary Figure 30.** GITT profiles of S@CA-$LiNO_3$ and S@$LiNO_3$ under the current density of 0.1 C.

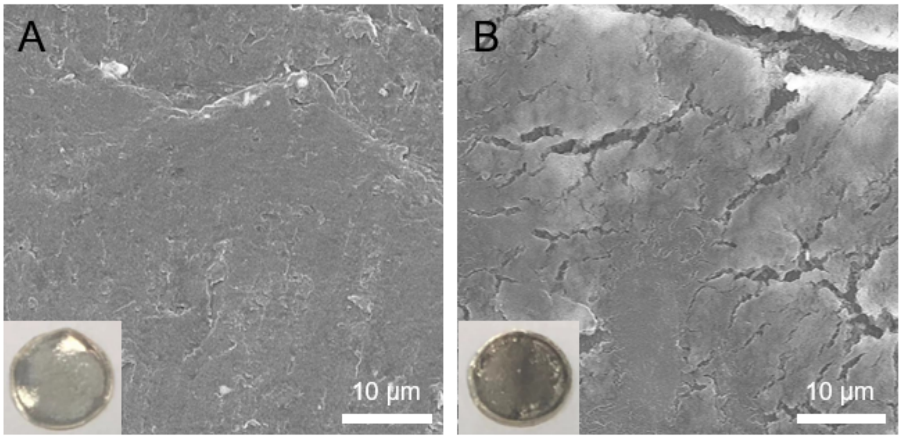


**Supplementary Figure 31.** SEM morphology of LMA after 500 cycles in (A) S@CA-$LiNO_3$ and (B) S@$LiNO_3$ under the current density of 1 mA $cm^{-2}$. (insert image: optical observation of LMA after cycling)

**Supplementary Table 1.** the LUMO and HOMO energy of solvent and electrolyte additives.

| | LUMO (eV) | HOMO (eV) |
|---|---|---|
| **DME** | -0.18205 | -7.18631 |
| **DOL** | -0.23946 | -7.22169 |
| **CA** | -2.14373 | -6.30547 |
| **LC** | -1.54181 | -5.88015 |
| **$LiNO_3$** | -1.59894 | -7.70355 |
| **LiTFSI** | -1.93773 | -9.16341 |

**Supplementary Table 2.** Performance comparison between Li@CA-$LiNO_3$ and other LMA in ether based electrolyte reported from literatures.

| | Maximum current density ($mA\ cm^{-2}$) | Cyclic stability of 1 $mAh\ cm^{-2}$ (cycles) | Cumulative capacity ($Ah\ cm^{-2}$) | Reference |
|---|---|---|---|---|
| **This work** | 10 | 4250 | 4.25 | |
| **TESM/Li** | 10 | 3000 | 3.5 | [1] |
| **APL** | 5 | 200 | 0.2 | [2] |
| **ND-Li** | 10 | 700 | 0.7 | [3] |
| **LiFSI/$LiNO_3$** | 0.5 | 500 | 0.5 | [4] |
| **$Li_{13}In_3$\|Li** | 2 | 1600 | 2.5 | [5] |
| **PS+$LiNO_3$** | 2 | 400 | 0.8 | [6] |
| **PST-90-electrolyte** | 2.25 | 1000 | 0.8 | [7] |
| **MFC-Li/CNF** | 10 | 300 | 1.53 | [8] |
| **NLi** | 8 | 1000 | 0.5 | [9] |
| **Li-LPS-PDMS** | 4 | 1000 | 0.84 | [10] |

## Supporting References


1 Ju Z., Nai J., Wang Y., Liu T., Zheng J., Yuan H., Sheng O., Jin C., Zhang W., Jin Z., Tian H. Biomacromolecules enabled dendrite-free lithium metal battery and its origin revealed by cryo-electron microscopy. *Nat. Commun.*, **11**, 1-10, (2020).

2 Xu R., Zhang X. Q., Cheng X. B., Peng H. J., Zhao C. Z., Yan C., Huang, J. Q. Artificial soft-rigid protective layer for dendrite-free lithium metal anode. *Adv. Funct. Mater.*, **28**, 1705838, (2018).

3 Zhang, W., Fan, L., Tong, Z., Miao, J., Shen, Z., Li, S., Chen, F., Qiu, Y., Lu, Y. Stable Li-metal deposition via a 3D nanodiamond matrix with ultrahigh Young's modulus. *Small Methods*, **3**, 1900325, (2019).

4 Zhang X. Q., Chen X., Hou L. P., Li B. Q., Cheng X. B., Huang J. Q., Zhang, Q. Regulating anions in the solvation sheath of lithium ions for stable lithium metal batteries. *ACS Energy Lett.*, **4**, 411-416, (2019).

5 Liang X., Pang Q., Kochetkov I. R., Sempere M. S., Huang H., Sun X., Nazar L. F. A facile surface chemistry route to a stabilized lithium metal anode. *Nat. Energy*, **2**, 17119, (2017).

6 Li W., Yao H., Yan K., Zheng G., Liang Z., Chiang Y. M., Cui Y. The synergetic effect of lithium polysulfide and lithium nitrate to prevent lithium dendrite growth. *Nat. Commun.*, **6**, 1-8, (2015).

7 Li G., Gao Y., He X., Huang Q., Chen S., Kim S. H., Wang D. Organosulfide-plasticized solid-electrolyte interphase layer enables stable lithium metal anodes for long-cycle lithium-sulfur batteries. *Nat. Commun*., **8**, 1-10, (2017).

8 Ren Y. X., Zeng L., Jiang H. R., Ruan W. Q., Chen Q., Zhao T. S. Rational design of spontaneous reactions for protecting porous lithium electrodes in lithium-sulfur batteries. *Nat. Commun.*, **10**, 1-10, (2019).

9 Li S., Fan L., Lu Y. Rational design of robust-flexible protective layer for safe lithium metal battery. *Energy Storage Mater.*, **18**, 205-212, (2019).

10 Pang Q., Zhou L., Nazar, L. F. Elastic and Li-ion–percolating hybrid membrane stabilizes Li metal plating. *Proc. Nat. Acad. Sci*., **115**, 12389-12394, (2018).
